# Chikungunya in Military Health System Beneficiaries, 2020–2024

**Published:** 2026-02-24

**Authors:** Shauna L. Stahlman, Kiara D. Scatliffe-Carrion, Charles E. McCannon

**Affiliations:** Defense Health Agency, Public Health Directorate, Armed Forces Health Surveillance Division, Epidemiology and Analysis Branch, Silver Spring, MD: Dr. Stahlman; Defense Health Agency, Defense Centers for Public Health–Aberdeen, MD: Ms. Scatliffe-Carrion, Dr. McCannon

Chikungunya is a mosquito-borne viral disease that can cause severe joint pain, fever, and other short- or long-term symptoms. ^1^ Chikungunya is endemic to tropical and subtropical regions, with cases and outbreaks recorded in more than 100 countries. ^2^ The U.S. Food and Drug Administration (FDA) recently approved 2 chikungunya vaccines: a live-attenuated vaccine called IXCHIQ in November 2023, and a virus-like particle vaccine called VIMKUNYA in February 2025. ^3^ These vaccines are recommended for those traveling to high-risk areas. The FDA recently suspended the U.S. license for IXCHIQ in August 2025, however, citing vaccine safety concerns. ^4^

This analysis was conducted to answer questions from military health leadership about the risk of chikungunya infection to service members and their families. The analysis employed data published in prior *MSMR* articles ^5 - 7^ to provide case counts for all Military Health System (MHS) beneficiaries from 2020 through 2024. Data were drawn from the Defense Health Agency's Disease Reporting System internet (DRSi), and were confirmed via medical chart review.

Ten cases of chikungunya virus disease among MHS beneficiaries were documented from 2020 through 2024 **(Table)** . Five cases were recorded in service members, 3 among family members (all spouses), and 2 in other beneficiary types (i.e., not service members or dependents). One case was acquired while on deployment to multiple locations in Southeast Asia; no other cases were related to official travel or deployment. Most cases were related to unofficial travel.

**TABLE:** Chikungunya Cases, Military Health System Beneficiaries, 2020–2024

Year of Onset	Beneficiary Type	Location of Travel	Deployment-related	Hospitalized ^a^	Long-Term Symptoms ^b^	Short-Term Symptoms (summarized)
2020	Family member (spouse)	Costa Rica	No	No	Yes	Fever, polyarthralgia, rash
2020	Other	Niger	No	N/A	N/A	N/A
2020	Other	Cameroon	No	N/A	N/A	N/A
2022	Navy active duty	Brazil	No	Yes	Yes	Fatigue, myalgia, rash, polyarthralgia
2022	Army National Guard	Mexico	No	No	No	Rash, polyarthralgia, myalgia, gait disturbance
2023	Air Force active duty	Indonesia	No	No	No	Influenza-like symptoms, headache, polyarthralgia
2023	Family member (spouse)	Guatemala	No	No	No	Rash, polyarthralgia
2023	Navy active duty	Southeast Asia (multiple countries)	Yes	No	No	Fever, headache, polyarthralgia
2024	Family member (spouse)	No travel around time of diagnosis	No	Yes	No	Rash, polyarthralgia, swollen lymph nodes
2024	Army active duty	Chad	No	No	No	Patient also had malaria; fever, headache, myalgias, nausea, vomiting, dysuria

Abbreviation: N/A, not available.

a Hospitalization likely related to chikungunya.

b Long-term symptoms defined as symptoms lasting longer than 12 weeks likely due to chikungunya.

Polyarthralgia, or pain in multiple joints, was the most documented symptom (n=7). Other commonly reported symptoms included fever, rash, and myalgia. Two cases had long-term symptoms (i.e., lasting longer than 12 weeks), and 2 cases were hospitalized. No cases had evidence of prior chikungunya vaccination in their medical records.

The small number of cases, hospitalizations, and evidence of long-term symptoms reported in the past 5 years suggest that risk of chikungunya virus disease to MHS beneficiaries is small. Use of standard preventive measures including personal protective equipment and vaccination should, however, continue to be encouraged when indicated for service members and other beneficiaries traveling to high-risk areas.
